# Dynamic stability of salt stable cowpea chlorotic mottle virus capsid protein dimers and pentamers of dimers

**DOI:** 10.1038/s41598-022-18019-9

**Published:** 2022-08-22

**Authors:** Janos Szoverfi, Szilard N. Fejer

**Affiliations:** 1 Faculty of Chemical Engineering and Biotechnologies, University Politehnica of Bucharest, 1-7 Gheorghe Polizu Street, 011061 Bucharest, Romania; 2 Provitam Foundation, 16 Caisului Street, 400487 Cluj-Napoca, Romania; 3 Institute of Chemistry, University of Pécs, 6 Ifjúság Street, Pécs, Hungary

**Keywords:** Self-assembly, Computational models

## Abstract

Intermediates of the self-assembly process of the salt stable cowpea chlorotic mottle virus (ss-CCMV) capsid can be modelled atomistically on realistic computational timescales either by studying oligomers in equilibrium or by focusing on their dissociation instead of their association. Our previous studies showed that among the three possible dimer interfaces in the icosahedral capsid, two are thermodynamically relevant for capsid formation. The aim of the current study is to evaluate the relative structural stabilities of the three different ss-CCMV dimers and to find and understand the conditions that lead to their dissociation. Long timescale molecular dynamics simulations at 300 K of the various dimers and of the pentamer of dimers underscore the importance of large contact surfaces on stabilizing the capsid subunits within an oligomer. Simulations in implicit solvent show that at higher temperature (350 K), the N-terminal tails of the protein units act as tethers, delaying dissociation for all but the most stable interface. The pentamer of dimers is also found to be stable on long timescales at 300 K, with an inherent flexibility of the outer protein chains.

## Introduction

Virus-like particles (VLPs) are becoming an increasingly important tool for a variety of applications in biotechnology and medicine. In order to be able to create efficiently self-assembling VLPs, however, it is necessary to understand the processes that stabilize these particles and guide their self-assembly. We do not have such an understanding at the atomistic level yet, and the number of publications about modelling association of peptides^1,2^ or formation of protein–protein complexes^3–5^ on an atomistic scale is surprisingly small. The formation of protein–protein dimers can be explained as a diffusive association succeeded by the formation of a stereospecific complex^6^. The cowpea chlorotic mottle virus (CCMV) capsid is an ideal system to study atomistically, as multiple copies of the capsid protein are able to efficiently self-assemble into VLPs even in the absence of genetic material, while the self-assembly process itself is hierarchical and well-studied experimentally^7–9^. It has been shown previously that for the whole CCMV capsid, the disassembly pathway is not a mirror of the assembly pathway^10^. Coarse-grained Monte Carlo simulations coupled with experiments show that in case of CCMV, the thermal dissociation is analogous to a two-dimensional phase transition, during which the dimer subunits on the capsid are in equilibrium with free dimers in solution^11^. Conformational stabilities of monomers in pentameric and hexameric environments in the salt stable CCMV icosahedral capsid have been modelled previously as well^12^. However, a better understanding of the dissociation process of CCMV dimers can give new insight into the dynamic stability of various dimers.

Several research groups revealed the importance of conformational fluctuations and solvation changes in the process of association and dissociation of proteins^13,14^. All-atom simulations could reveal significant moments in the process of complex formation or dissociation, but due to the time and resource cost of such calculations just a few publications have been released in this topic. A study by Zhang et al.^15^ seeks to understand the dissociation of protein complexes through long time all-atom molecular simulations of two mutants, these are compared to previous simulations of the wild-type protein by the same group^16^. The majority of simulations showed that protein dissociation occurred within $2.4~\upmu \hbox {s}$. After the analysis of the solvation energies Zhang et al. observed that the dissociation correlates with loss of protein–protein contacts in the complex. Thermodynamics of the separation is influenced significantly by the internal protein dynamics. The final conclusion of the research shows that the process of dissociation usually follows multiple pathways and involves transitions to less favorable interface contacts. Banerjee et al. analyzed the contribution of the dynamics of the water in the dissociation or association process of insulin monomer^17^. They achieved the separation of insulin dimers and showed that water molecules play an important role in the separation or association of proteins.

CCMV is an icosahedral plant virus with 12 pentameric and 20 hexameric capsomers, with a triangulation number of 3^18^. The coat protein subunits are comprised of an eight stranded, antiparallel, $\beta$-barrel core. The capsomers are probably stabilized by the linkage through the extensions of the C-terminal from coat proteins^18^. The N-terminal region from one protein clamps the adjacent protein’s C-terminal arm. It is thought that a hexameric tubular structure, called $\beta$-hexamer, plays an important role in the stability of the capsid through the the N- and C-terminal extensions of the coat proteins contributing to the formation of the quaternary structure, while the hexameric tubular structure allows for tight clustering of neighbouring monomers into capsomer structures^18^. Chen et al. studied the interactions between the components of the CCMV capsid^19^ and found that flexible N-terminal tails are not only involved in the formation of protein dimers, but responsible for kinetic traps as well. The observed stability of the pentamer and hexamer of dimers suggests that the dimers themselves serve as the basic building blocks throughout the capsid formation process. A point mutation (K42R) in the N-terminal tail increases the capsid stability under high ionic strength conditions (salt stable CCMV)^20,21^.

**Figure 1:**
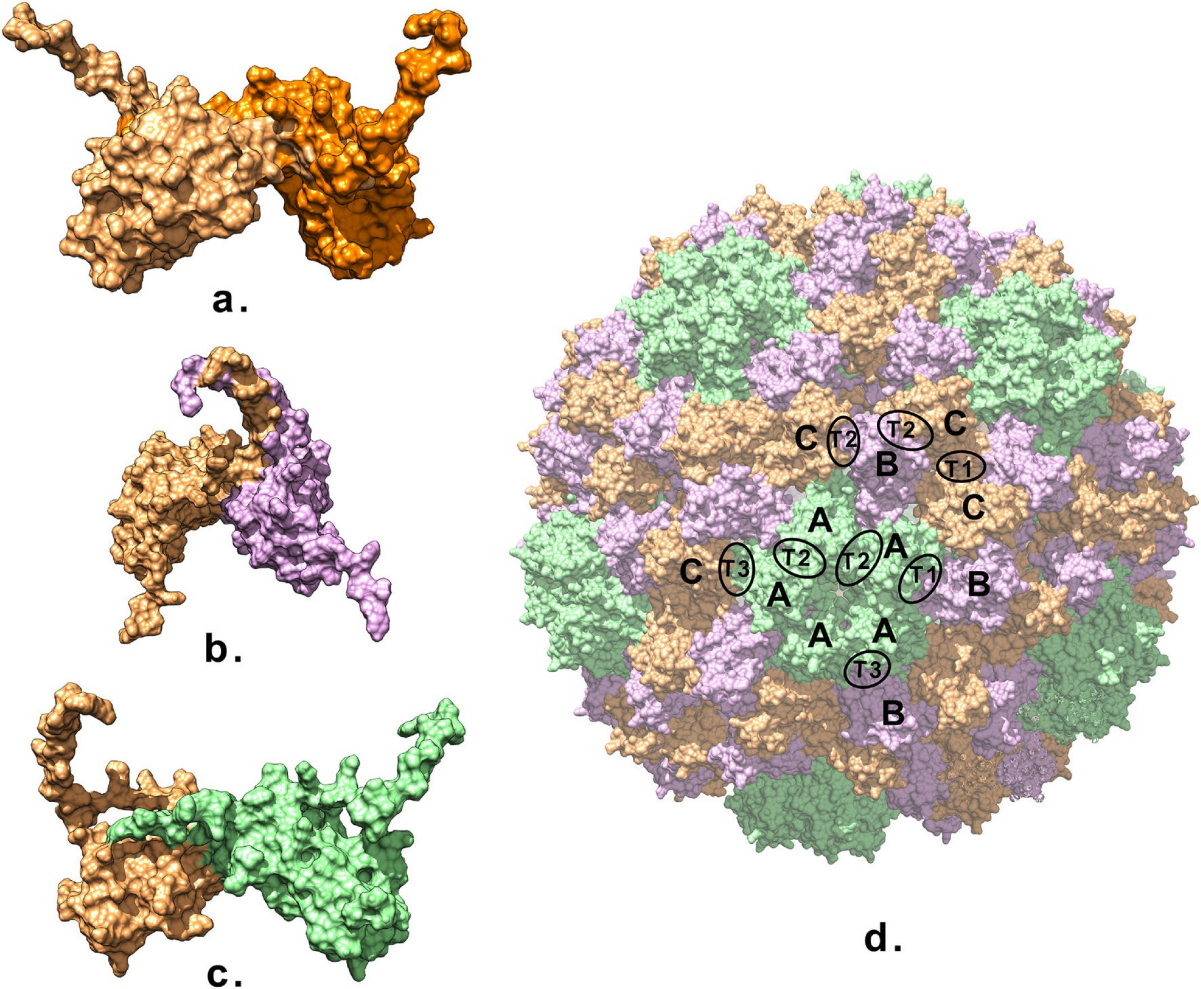
(**a**) T1, (**b**) T2 and (**c**) T3 dimers from the original capsid; (**d**) the whole icosahedral ss-CCMV capsid, coloured by chains, showing the positions of the three interface types between the protein monomers.

Our aim was to investigate the dynamic stability of oligomers that can act as building blocks for the salt stable CCMV (ss-CCMV) capsid^21^, namely the changes happening on the protein–protein interface. We performed in silico simulations under different conditions to achieve the dissociation or destabilization of protein dimers in various configurations compatible with the icosahedral ss-CCMV capsid architecture. All-atom molecular dynamics simulations were performed in implicit and explicit solvent at different temperatures to understand in detail the processes that could lead to the dissociation of a dimer, and, implicitly, give us insight into the relative dynamical stabilities of the various dimers. The simulated dimers were analyzed with respect to changes in the binding interface of the proteins (Fig. 1). A pentamer of dimers was selected from the icosahedral virus capsid and simulated in similar conditions as the dimers, in order to gain information about the behaviour of interfaces in a more restricted environment.

## Results and discussion

### Long timescale MD simulation for T1, T2 and T3 interfaces

Three dimers with different types of interfaces were selected from the ss-CCMV protein capsid (PDB id 1za7, 165 residues) to study their behaviour during all-atom MD simulation in explicit water. Dimers from the original, icosahedral virus capsid were used to characterize the initial interfaces using the Pymol InterFaceResidue plugin and the CoCoMaps webserver^22^, with interaction distance cutoff set at 5 Å^23^. Note that this is an inter-residue cutoff distance, so two residues are defined to be in contact whenever any two atoms belonging to the different residues are closer than this cutoff. The types of interactions are defined by CoCoMaps according to the polar nature of the residues in contact: hydrophobic residues are non-polar (e.g. Ala, Val, Leu, Ile, Thr), hydrophilic residues have polar sidechains (e.g. Glu, Asp, Ser, Thr, Asn).

As the first 25 residues are missing from the 1za7 pdb structure for chains B and C, in order to have continuous numbering for the dimer structures, we restarted numbering of the chains starting from residue 26. Our numbering scheme therefore goes from 1 to 330 for the dimers. Note that for chain A, 39 residues could not be resolved in the crystal structure, so we constructed the three possible dimer interfaces from chains B and C.

The T1 dimer has the largest interface between the three types, with an interface surface area of 2957.6 Å$^2$, 14% of the total surface area of two monomers, and it involves 40 residues out of 330. The interactions stabilizing the connection of the monomers are mainly hydrophilic–hydrophilic interactions (36), hydrophobic–hydrophobic interactions (40) and hydrogen bonds (18). The stabilizing hydrophilic–hydrophilic interactions have a polar character, an electronegative atom (oxygen or nitrogen) being in contact with a hydrogen atom in a position that does not satisfy criteria for hydrogen bonds. The dimer has $C_2$ rotational symmetry, the angle between the two monomers is $72^\circ$. The interface surface area of T2 dimer is 2034.7 Å$^2$, 9.8% of the total surface area of two monomers. 31 residues are involved in this interface. The angle between the two monomers is $34.6^\circ$. There are 39 hydrophobic–hydrophobic interactions, 12 stabilizing hydrophilic–hydrophilic interactions and 12 hydrogen bonds between the monomers.

The T3 dimer has 13 residues on the interface. The buried area upon complex formation is 838 Å$^2$, 4.05% of the total surface area of two monomers. 16 stabilizing hydrophilic–hydrophilic interactions, 4 hydrophobic–hydrophobic interactions and 3 hydrogen bonds are identified between the monomers.

$2~\upmu \hbox {s}$ NPT ensemble molecular dynamics simulations were carried out at 300 K for T1, T2 and T3 dimers (see Supplementary Movies 1–3). The simulations for T1 and T2 were repeated with different initial velocities, but as the results were similar for the first microsecond, the process was stopped to save computational time. The complete trajectories for all explicit solvent simulations of T1 and T2 are shown in Supplementary Fig. 7. RMSD calculations were carried out for the $\mathrm {C}_\alpha$ backbone of the proteins and for the binding interface residues identified in the 1za7 PDB structure.

**Figure 2:**
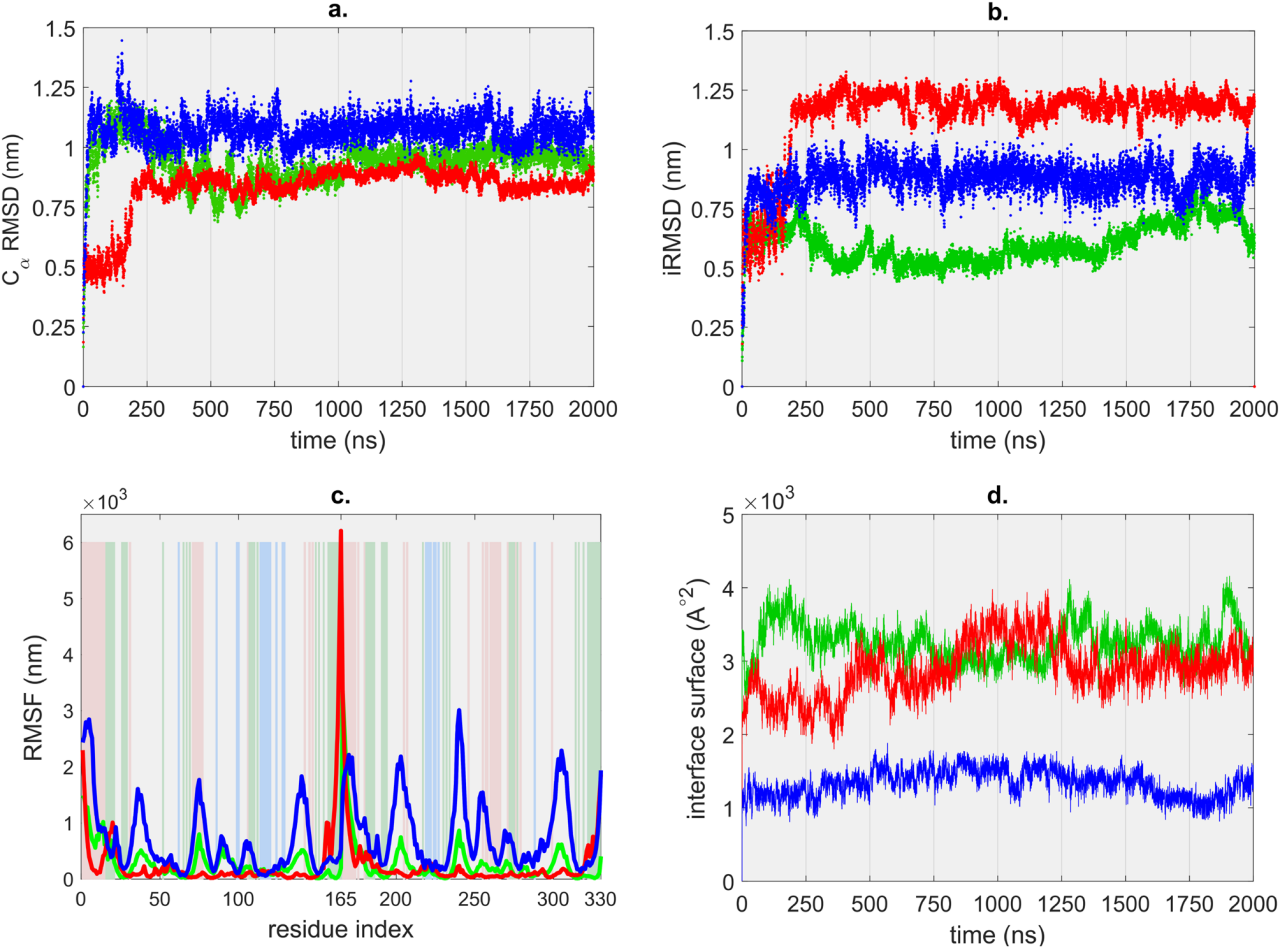
(**a**) $\mathrm {C}_\alpha$ RMSD, (**b**) interface RMSD, (**c**) per-residue fluctuation (background colour bars represent the interface residues for T1 (green), T2 (red), T3 (blue), (**d**) change of the interface surface area during the trajectory for T1 (green), T2 (red) and T3 (blue) dimers.

$\mathrm {C}_\alpha$ RMSD values for T1 show a bigger fluctuation during the simulation and reach a value of about 1.1 nm in the first 300 ns, stabilizing after that at around 0.9 nm (average $0.94 \pm 0.09~\hbox {nm}$). $\mathrm {C}_\alpha$ RMSD of T2 grows to an average value of $0.82 \pm 0.1\,\hbox {nm}$. The average $\mathrm {C}_\alpha$ RMSD of the T3 dimer is $1.1 \pm 0.73 \,\hbox {nm}$, having an accentuated fluctuation compared to that of T1 or T2 (Fig. 2a).

The tail regions of the dimers have specific roles in the stabilization of the dimers and the formation of the capsid^24^. In both the T1 and T2 dimers the regions with the highest movement are the terminal regions, but their contribution to the $\mathrm {C}_\alpha$ RMSD is different for T1 and T2, due to their position. In case of the T1 dimer, the C-terminal residues are located mostly on the interface between the monomers, while the N-terminal residues can move more freely. The terminal regions of the T2 dimers are located in the outer regions of the protein dimer. The average $\mathrm {C}_\alpha$ RMSD value calculated for the dimers without the flexible tail regions is $0.71 \pm 0.1~\hbox {nm}$ for T1 and $0.58 \pm 0.08~\hbox {nm}$ for T2 (Fig. 2a). The $\mathrm {C}_\alpha$ RMSD value of 0.71 nm for the main body of the T1 dimer is remarkably similar to that of previous 500 ns long simulation results of wild type CCMV dimers^19^.

The interface RMSD (iRMSD) values calculated for each trajectory show the movement of atoms from the residues present on the contact area between the two proteins in each dimer (Fig. 2b). The average iRMSD for T1 ($0.6 \pm 0.08~\hbox {nm}$) is much lower than that for T2 ($1.14 \pm 0.15~\hbox {nm}$). This indicates that the T2 interface is structurally less stable than T1, this being in a good agreement with our previous study^25^. Within the first 50 ns of the simulation the values quickly increase to over 0.7 nm. Interface RMSD values for the T1 dimer show a deviation from the average between 1500 and 1950 ns, afterwards returning to the average values. The change is caused by a temporary large-scale movement of the hairpin between residues 10 and 24, away from the body of the protein. We continued the simulation only for this trajectory to a total length above $2.4~\upmu \hbox {s}$ and found that the average iRMSD of 0.6 nm does not change within this timeframe for the T1 interface (see Supplementary Fig. 3).

The N-terminal tail regions of the monomers are in close contact in the T2 dimer and are located away from the body of the monomers, therefore the tail regions are quite mobile. After 200 ns simulation this tail gets glued to the main body of the dimer and remains there for the time of the simulation, while the interface between the two tails is also completely reorganised. Due to these changes, the iRMSD of the T2 dimer reaches very high values (avg $1.14 \pm 0.16~\hbox {nm}$), compared to that of the starting structure, which wrongly suggests an accentuated instability of the interface. For this reason, the iRMSD was recalculated without the first 14 residues from the N-terminal (residues missing from the A chain of the protein asymmetric unit in the PDB structure), and we found that the contribution of the N-terminal tail to the iRMSD is around 30%.

Per-residue fluctuations were calculated to monitor the motion of the residues (Fig. 2c). Interface residues show a higher mobility for the T1 dimer due to the fact that several exposed residues are part of the terminal regions. Regions containing $\beta$-sheets from the core have a lower mobility, although the outer turns present bigger motions. Tail regions show much higher values both on the N- and C-terminal parts of the monomers (see Supplementary Fig. 1) . Several residues of the $\beta$-barrel are on the protein–protein interface. The high iRMSD values observed for T2 are due to the accentuated movement of tail regions, as more such residues are located on the interface. In case of the T3 dimer the overall fluctuation is bigger than for the others, as the relative orientation of the chains and the small interface allow for bigger motions. The N-terminal tails are located away from the body of the dimer, and are not involved in the forming of the interface, so they can move freely, shown by the high fluctuation values. The peaks shown in Fig. 2c between those for the terminal regions represent motions of the turns from the $\beta$-barrels in the outer region of the dimer, these are most pronounced for T3.

The solvent accessible surface area (SASA) of the dimers is relatively constant throughout the simulation for each dimer. In our case the examination of the binding surface is more relevant. The interface surface was calculated as the difference of the dimer SASA and that of the standalone monomers and is shown in Fig. 2d. In all cases the interface surface change through each trajectory shows a slow increase with high fluctuation. The interface surface of the T1 dimer is initially higher than that of the T2 dimer, increasing from the initial 14% of the total surface to 19%. The large oscillation of the values shows that the solvent accessibility of the interface changes continuously due to small-scale movements. The surface of the T2 interface is smaller but after 1000 ns of simulation increases to that observed for T1 with an accentuated increase between 800 and 1200 ns. The interface surface of T3 stays relatively constant with low oscillation of the values.

The behaviour of the monomers relative to each other was investigated by tracking the change in distances, number of contacts (polar and non-polar interactions), number of hydrogen bonds, and the angles defined by the principal axis of inertia of the monomers.

The distance between the center of mass of the monomers in case of T1 is constant ($3.8 \pm 0.13~\hbox {nm}$) with a temporary decrease between 400 and 700 ns. Both the number of contacts ($293 \pm 34$) and the hydrogen bonds ($25 \pm 4$) shows a slow increase. However, we observe a significant variation during the first 1000 ns in the angle between the two monomers, decreasing to $33^\circ$ and subsequently increasing to $90^\circ$, eventually oscillating around the original $70^\circ$ for the rest of the trajectory. This fan-like motion was previously described by Globisch et al.^26^.

As we did not observe drastic changes on the interfaces of three types of dimers during the long simulations, we chose another, putative dimer from the docking results of our previous work^25^ that had the biggest RMSD score (3.09 nm) when compared to the original T3 dimer in order to start the simulation from a dimer that is not present in the icosahedral capsid and is therefore far from any native protein-protein interface for ss-CCMV. The chains in this dimer (type X, TX) are bound together through the first 15 residues of the N-terminal tail. Molecular dynamics simulations were carried out under the same conditions than for the other dimers (see Supplementary Movie 4). As only the N-terminal tails are in contact in the TX dimer, the flexibility of the dimer is rather high (see Supplementary Fig. 4). The fluctuation of the residues is substantially larger than for the other 3 (native) dimers. After 100 ns of simulation the $\mathrm {C}_\alpha$ RMSD reaches 1.4 nm and stabilizes around this value. The monomers are coming closer to each other and at a moment they are forming contacts between the turn regions of the $\beta$-barrel cores. The N-terminal tails remain in close contacts and stick to the body of each monomer. The number of contacts and hydrogen bonds increase constantly through the simulation. At the starting point of the simulation only 8 hydrogen bonds are found, mainly between the N-terminal tails. During the simulation an average of $16 \pm 3$) bonds are present, with new connections being formed between the loop regions of $\beta$-barrels and the tails and the body of dimers. The distance between the monomer chains is reduced from 4.5 to 3.9 nm and the angle between them from $90^\circ$ to $10^\circ$. The structural stability of the TX dimer is therefore much lower than that of any native (T1, T2, T3) dimers.

In order to assess the possible mechanistic reasons for the enhanced stability of ss-CCMV compared to the wild type CCMV capsid, we analyzed the contacts and hydrogen bonds of the arginine residue located at the point mutation site of ss-CCMV (ARG17—chain C, ARG182—chain B). Note that ARG17 and ARG182 are at position 42 of chains C and B of the ss-CCMV protein when using the original numbering convention (see Supplementary Fig. 2). Throughout the $2~\upmu \hbox {s}$ NPT simulations, the arginine residues of the T1 dimer have the most contacts: ARG17 is in contact with PRO328 (for $0.52~\upmu \hbox {s}$ in total along the trajectory), has a hydrogen bond with ASP324 ($0.14~\upmu \hbox {s}$); ARG182 contacts with PRO163 ($1.98~\upmu \hbox {s}$), VAL164 ($1.82~\upmu \hbox {s}$), TYR165 ($1.86~\upmu \hbox {s}$), has a hydrogen bond with TYR165 ($0.2~\upmu \hbox {s}$). In the T2 dimer, ARG17 is in contact with PRO279 ($1.0~\upmu \hbox {s}$), GLU280 ($0.6~\upmu \hbox {s}$), has a hydrogen bond with GLU316 ($0.4~\upmu \hbox {s}$); ARG182 has a hydrogen bond with GLU9 ($0.4~\upmu \hbox {s}$). For the T3 dimer, ARG17 has no contacts, while ARG182 contacts only with TYR165 ($1.8~\upmu \hbox {s}$). It is therefore possible that the point mutation stabilizes the T1 dimer the most, compared to the T2 and T3 dimers. However, in order to better assess the possible reason for structure stabilization, contact environments should be directly compared between wt-CCMV and ss-CCMV. Unfortunately, the lower resolution of the wt-CCMV crystal structure (PDB ID 1cwp) and the difference in missing residues complicates such attempts. A more straightforward way to compare the two environments would be to use the optimised ss-CCMV dimer structures as templates, reverting the mutation to wild type, reoptimising the structures and redoing the long simulations.

### Behaviour of T1, T2, T3 and TX interfaces at 350 K

**Figure 3:**
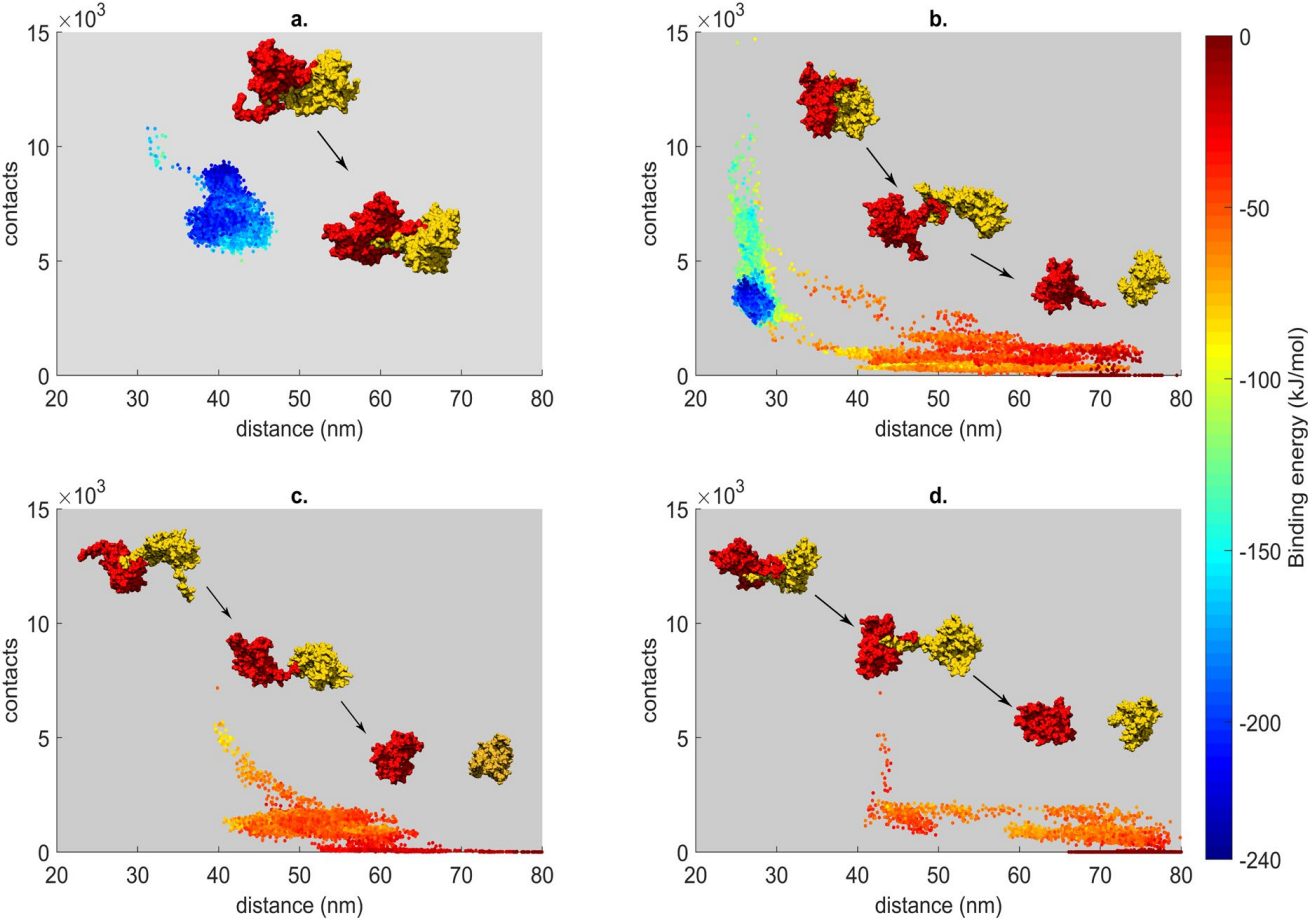
Binding energy landscape as a function of monomer contacts and center of mass distances, with initial and final structures presented, of the implicit water MD simulations for T1 (**a**), T2 (**b**), T3 (**c**) and TX (**d**) at 350 K. All frames from the four trajectories per dimer were used to generate these graphs.

A remarkable property of the wild type CCMV capsid is that it is stable at $75\,^\circ \mathrm {C}$ at pH 4.5 and ionic strength of 0.1 M^27^. We chose a temperature slightly above this temperature to see if dissociation is possible in the three dimer configurations, as during capsid disassembly some protein–protein interfaces must disappear. No experimental data is available for the melting temperature of the ss-CCMV capsid. However, all three brome mosaic virions (a virus related to CCMV) have remarkably similar melting temperatures to that of wild type CCMV (about $70\,^\circ \mathrm {C}$)^28^. We expect similar dissociation/melting temperatures for ss-CCMV as well. Note that capsid melting does not necessarily mean complete dissociation into monomers in experiments, so melting and dissociation temperatures are not necessarily equivalent. As these temperatures depend strongly on pH as well, simulations have to aim higher than the highest experimental temperature to achieve protein–protein dissociation that can destabilize a fully formed capsid. We therefore performed 4 parallel molecular dynamics simulations at 350 K in implicit solvent for each of the T1, T2, T3 and TX dimers to see which interface can be most destabilized in the absence of explicit water molecules. The simulations had the same starting structures, and only differed in the initial velocities, while other parameters were similar to those in the simulations with explicit water. The T1 dimer proved to be relatively stable a this temperature (Fig. 3a), with a bigger fluctuation of the residues ($\mathrm {C}_\alpha$ RMSD of $0.99 \pm 0.11~\hbox {nm}$) and minor changes on the interface (iRMSD of $0.61 \pm 0.1~\hbox {nm}$). The biggest contribution to the overall movement is given by the N-terminal tail that can move freely at the beginning of the simulation.

In case of the other three dimers (T2, T3 and TX), dissociation events occurred in at least one of the four parallel runs (Fig. 3b–d, see also Supplementary Movie 5). We observed two different mechanisms for dissociation of the dimers at 350 K. Complete dissociation took place swiftly in one simulation, while in the three other trajectories the dimer reached a near-dissociated state during the first 5–10 ns of the simulation. The interaction between the N-terminal tails still maintains a small interface, while the monomers keep moving away but stay hooked together at the same time through this small interface (see Supplementary Fig. 5). By 75 ns the dissociation of the dimer is complete in these trajectories as well. While both the SASA and the interface surface is slowly increasing for T1 throughout the simulation, we observe an increase of SASA and a large decrease of interface surface for three of the four simulations just before complete dissociation (Supplementary Movie 5) or the formation of the tether between the two tails. In case of the remaining 8 trajectories in total for T2, T3 and TX, the N-terminal tails of the dimers remain attached and prevent the complete separation of the two chains throughout the simulations. Supplementary Figure 6e shows the last frames of each high-temperature trajectory in which dissociation is not complete.

The N-terminal tails act as a tether and prevent dissociation in three different dimer configurations (T2, T3 and TX), suggesting that this large, mobile part of the protein can in fact play an important role during the self-assembling process, increasing the effective concentration of interacting building blocks by forming weakly bound flexible intermediate states and allowing the chains to find the most favourable native contacts. As the RNA binding domain (residues 1–25) is missing from the ss-CCMV structure used here, the real N-terminal region is considerably longer, and therefore the tethering effect aiding assembly of empty capsids in vitro might be important on double the length scale observed in our simulation. As the N-terminal domain is essential for RNA binding, such a tethering effect is likely important for the assembly of *empty* capsids only, in solutions with no RNA present, speeding up the association of T1 dimers into the pentamer of dimer seed structure, and helping the subsequent addition of T1 dimers to the seed.

**Figure 4:**
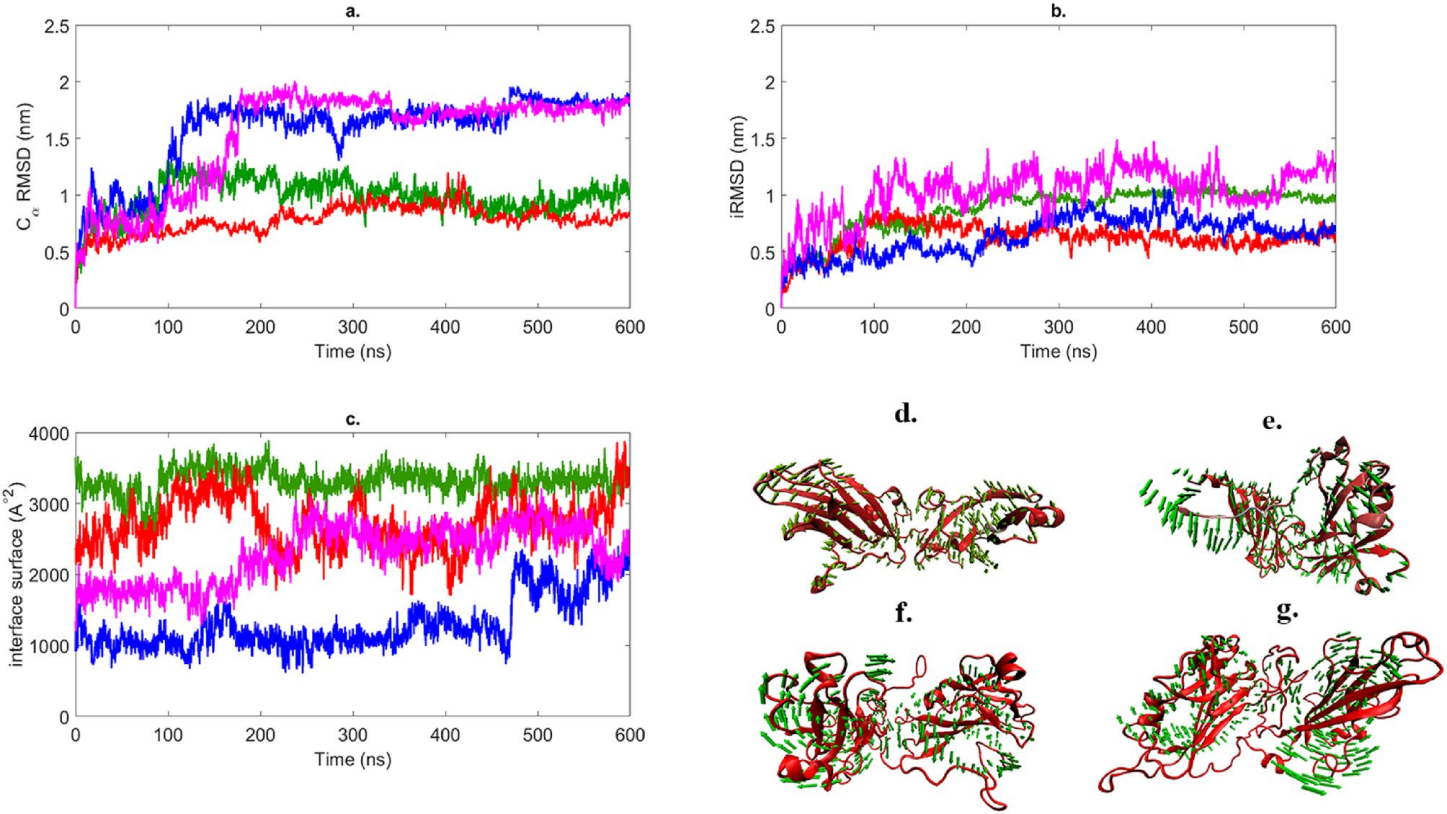
(**a**) $\mathrm {C}_\alpha$ RMSD, (**b**) iRMSD and (**c**) interface surface for T1 (green), T2 (red), T3 (blue) and TX (purple) for 600 ns NPT ensemble in explicit water on 350 K. Structures (**d–g**) show the lowest frequency motions for T1 (**d**), T2 (**e**), T3 (**f**) and TX (**g**).

After we observed that in certain conditions the dissociation of dimers is possible, we decided to inspect the behaviour of the dimers at 350 K in explicit water and constant pressure. However, during the 600 ns simulations all dimers remained associated (Fig. 4). The T2 dimer presents $\mathrm {C}_\alpha$ RMSD value of 0.64 nm, lower than that observed at 300 K. In contrast, T3 and TX have high $\mathrm {C}_\alpha$ RMSD values at 350 K. These two dimers are attached mostly through their N-terminal tails with relatively small binding interfaces, therefore an accentuated motion of the protein chains is possible. The interface surface area is growing in all simulations of the T2, T3 and TX dimers. We performed normal mode analysis for the four simulations (Fig. 4d–g). The low-frequency mode of the T1 dimer shows the fan-like motion described previously with minor loss in secondary structure. The T2 interface presents a twisted relative motion of the subunits, with an accentuated motion of the terminal residues. The monomers of the T3 dimer are moving more independently to each other, the lowest mode being similar to a rotatory motion of one of the chains. The character of motions is similar in case of the TX and T3 interfaces, the TX interface changes continuously during the simulation.

Although the different behaviour of high temperature simulations in explicit and implicit solvent is likely due to multiple factors (simulations are being done on essentially different energy landscapes due to the different solvent models), the inherent viscosity of explicit solvent models is an important factor hindering dissociation. The dimer in implicit solvent simulations does not have to ‘push away’ water molecules, and that can explain why the T2, T3 and TX dimers readily dissociate. Explicit and implicit models have been compared previously by Anandakrishnan et al.^29^ and it was found that the implicit model can be up to 100-fold faster than the explicit model for reproducing large-scale conformational changes.

### Long timescale MD simulation for the pentamer of dimers

**Figure 5:**
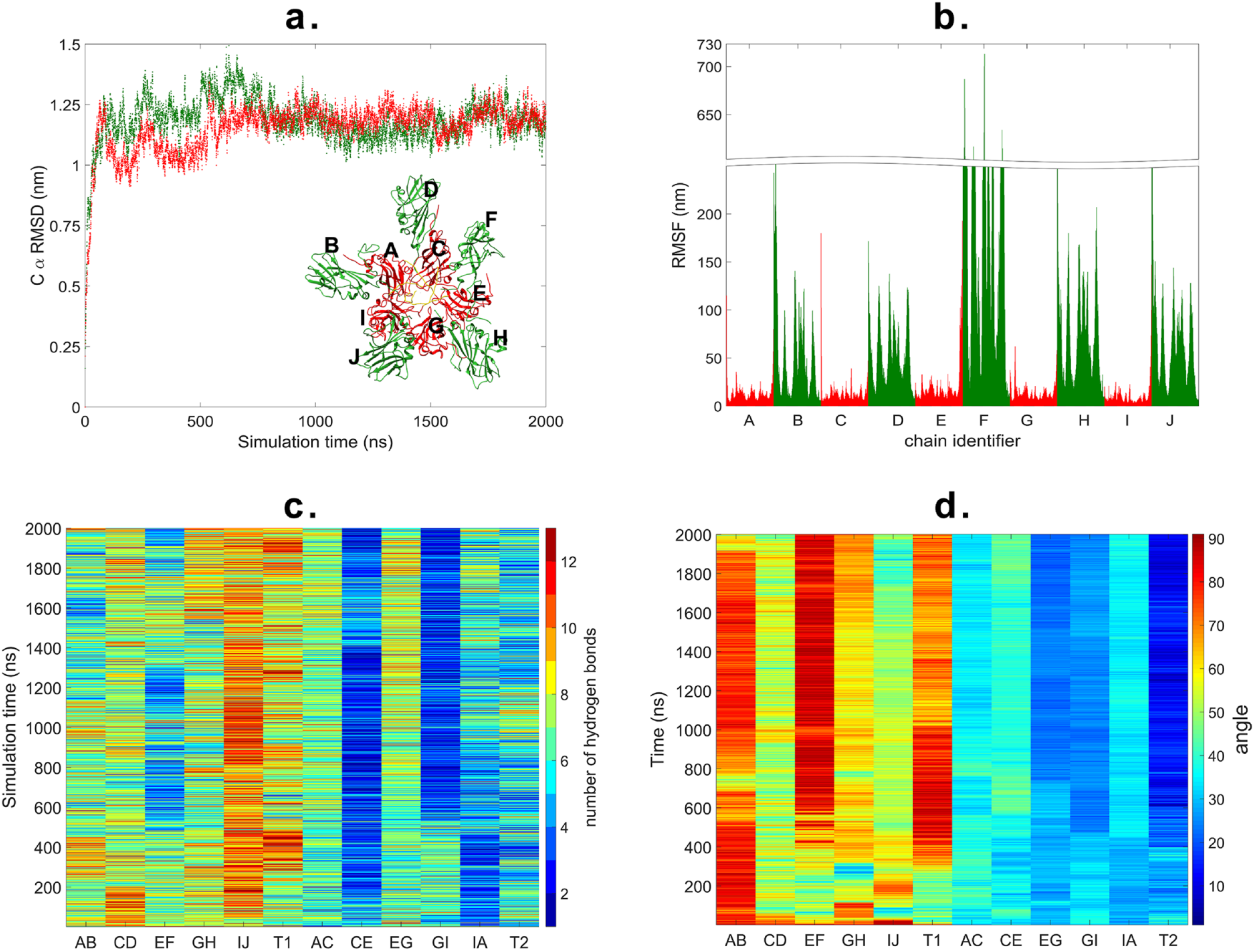
(**a**) $\mathrm {C}_\alpha$ RMSD of the PD from two parallel MD simulations starting from different random velocities from the same structure for $2~\upmu \hbox {s}$; (**b**) residue fluctuation in the PD for the inner chains (red) and outer chains (green); (**c**) variation in the number of hydrogen bonds for the separated T1 dimers (AB ... IJ), standalone T1 dimer (T1) and separated T2 dimers (AC ... IA), standalone T2 dimer (T2); (**d**) variation in the angle between the body of monomers in the separated T1 dimers (AB ... IJ), standalone T1 dimer (T1) and separated T2 dimers (AC ... IA), standalone T2 dimer (T2).

Trajectories of the two parallel $2~\upmu \hbox {s}$ simulations (10000 frames, 0.2 ns/frame) were analyzed, and are shown in Fig. 5a (see also Supplementary Movie 6). $\mathrm {C}_\alpha$ RMSD was calculated for both of the trajectories with reference to the first frame. The RMSD values grew rapidly in the first 30 ns of simulation, afterwards a fluctuation between 0.1 and 1.4 nm was observed with an average of $1.10 \pm 0.12~\hbox {nm}$ for the first run and $1.21 \pm 0.12~\hbox {nm}$ for the second run, respectively. Figure 5b shows the residue fluctuations of the inner and outer chains in the PD. Hydrogen bond and angle variations during the simulations are illustrated in Fig. 5c,d, respectively.

The pentamer of dimers (PD) is stable throughout the simulation, which is in good agreement with previous observations suggesting that the PD is a stable subunit during the assembly of the full icosahedral capsid^30^. For a better view of the changes during the simulation, we analysed the dimers contained in the PD by the interface type, as there are 5 dimers of T1 interface (AB-IJ) and 5 dimers of T2 interface (AC-IA) in this structure. The average $\mathrm {C}_\alpha$ RMSD and average interface RMSD values for the dimers within the PD are summarized in Table 1.

**Table 1:** Average $\mathrm {C}_\alpha$ RMSD and iRMSD for each dimer of the pentamer of dimers.

	$\mathrm {C}_\alpha$ RMSD (nm)	iRMSD (nm)
T1 AB	$0.77 \pm 0.07$	$0.29 \pm 0.04$
T1 CD	$0.79\pm 0.08$	$0.38 \pm 0.05$
T1 EF	$1.09\pm 0.19$	$0.69 \pm 0.18$
T1 GH	$0.89\pm 0.07$	$0.4 \pm 0.06$
T1 IJ	$0.82 \pm 0.07$	$0.34 \pm 0.05$
T2 AC	$0.3 \pm 0.03$	$0.21 \pm 0.02$
T2 CE	$0.36 \pm 0.04$	$0.26 \pm 0.05$
T2 EG	$0.35 \pm 0.04$	$0.21 \pm 0.02$
T2 GI	$0.42 \pm 0.06$	$0.32 \pm 0.05$
T2 IA	$0.28 \pm 0.03$	$0.21 \pm 0.02$

The large differences between the two types of interface is due to the fact that the T2 interfaces are located in the inner circle of the PD, thus their mobility is reduced. T1 interfaces are between a protein from the inner chains and another one from the outer circle. The outer chains have overall less contacts, therefore they are more mobile. Clearly, this mobility is present only in the PD configuration, and the whole PD structure must be as rigid as the inner part of the pentamer of dimers, when incorporated in the icosahedral capsid. The flexibility of the outer chains of the standalone PD might be useful to enable the subsequent addition of T1 dimers during the capsid assembly.

Among the T1 dimers, the one labelled EF behaves differently from the others. After 500 ns simulation an increase of 0.4 nm both for $\mathrm {C}_\alpha$ RMSD and iRMSD can be observed. The outer monomer moves out of the plane of the PD (see Supplementary Fig. 6). The movement is similar to the fan-like motion of the standalone T1 dimer. The N-terminal regions of the inner monomers are arranged in a specific conformation, forming hydrogen bonds with the tail regions of two neighboring chains on one side. The arrangement is conserved during the simulation, thus the role of the N-terminal region in the PD formation and stabilization is emphasized^24^. The N-terminal tails of the outer chains are sticking to the body of the monomer in the initial phase of the simulation.

To understand the movement of the protein chains, we calculated the angles between adjacent chains using as reference an axis for each of the monomer bodies (monomers without the terminal regions). The average angles for two dimers (AB, EF) were similar to those of the original T1 dimer, while the rest showed lower values. Our results suggest that the EF dimer from the PD is behaving like a standalone T1 dimer.

A small decrease in the number of hydrogen bonds can be observed for two dimers (AB, EF), while the other T1 dimers had similar values as for the standalone dimers. The average angles between the chains in T2 dimers are similar to those in the initial T2 dimer. The fluctuation of the values is different for the 5 dimers. The EG and GJ dimers behave similarly to the standalone T2 dimer.

## Conclusions

Our long molecular dynamics simulation results demonstrate the increased dynamic stability of the T1 interface compared to the other two native interfaces that exist in the icosahedral capsid configuration. The difference in stabilities becomes more evident at high temperature (350 K), where dimers connected by less stable, T2 or T3 interfaces, dissociate or remain only loosely associated via the flexible N-terminal regions being hooked together. Although proper dissociation is achieved only using implicit solvent models, the high-temperature explicit solvent simulations also point to the enhanced stability of the T1 interface compared to the other two.

We have also shown that an important assembly intermediate, the pentamer of dimers is a stable structure on the timescale of the simulation, with more flexible outer regions and possible fan-like motions localised on T1 dimers. The inner part of the PD is more rigid, while the flexibility of the outer part might be important in the next step of capsid self-assembly, the subsequent addition of T1 dimers to the PD seed.

## Methods

### Studied systems

Dimers of ss-CCMV capsid protein were selected according to our previous study^25^. The T1 and T2 interfaces play a significant role in the capsid formation, while T3 seemed less relevant. Type X is the dimer that had the highest RMSD compared to the original T3 dimer, selected from the 2000 docking results of two monomers.

Every dimer in the study has 4996 atoms in 330 residues. Identification of interfacial residues and characterization of the protein-protein interfaces in the three dimers was made using the InterfaceResidues Pymol script^31^. All structures were prepared with LEaP^32^. Solvation was done with an octahedral box of TIP3P^33^, where water molecules were added to a distance of 10 Å from the protein. The overall negative electrostatic charge was redistributed among all atoms in explicit water simulations. The systems contained the same number of waters and the total charge was neutralized with 6 added $\hbox {Na}^+$ ions.

A pentamer of dimers (PD) was selected from the capsid of ss-CCMV. The structure contains 24,980 atoms in 1650 residues. The inner part of the PD consists of 5 chains connected with the outer chains through T1 interfaces, while T2 interfaces are present between them. For a detailed analysis of the simulations, the PD was segmented into 10 dimers by the interface between the monomers: T1 interfaces (AB-IJ) and T2 interfaces (AC-IA). The PD was solvated with an octahedral box of TIP3P^33^ with water molecules to a distance of 10 Å from the protein. The system was neutralized with 30 $\hbox {Na}^+$ ions.

### Molecular dynamics simulations

The same MD protocol, adapted from Mafucci et al.^34^ was used for all NPT simulations.

- Hydrogen atoms were minimized for 1000 cycles of steepest descent and 5000 cycles of conjugated gradient, while the rest of the atoms were restrained.
- Water molecules and ions were relaxed using 2000 cycles of steepest descent and 5000 cycles of conjugated gradient.
- The solvent box was equilibrated at 300 K by 100 ps of NVT and 100 ps of NPT simulation using a Langevin thermostat and Berendsen barostat, respectively, with restraints on every atoms, excluding waters.
- The energy of side chains and water molecules was then minimized with backbone restraints of 25 kcal/mol.
- Complete minimization with backbone restraints of 10 kcal/mol (2500 cycles of steepest descent and 5000 cycles of conjugated gradient).
- Heating up the system to 300 K in 6 steps of 5 ps each ($\Delta$ T = 50 K), where backbone restraints were reduced from 10 kcal/mol to 5 kcal/mol.
- Full equilibration in the NVT ensemble (100 ps, backbone restraints of 5 kcal/mol) and in the NPT ensemble (1 step of 200 ps, backbone restraints of 5 kcal/mol; 3 steps of 100 ps each, reducing the backbone restraints from 5 to 1 kcal/mol, and 1 step of 1 ns with 1 kcal/mol of backbone restraints).

Molecular dynamics simulations were performed with the pmemd.cuda module of the Amber14 software package on NVIDIA Tesla K40 GPU. The force field used was ff03^35^. Pressure equilibration was made on manually edited systems by running NPT simulation several times on high pressure (1000 bars) for 20 ps until a density of $\approx 1.00~\hbox {kg}/\mathrm {dm^3}$, all atoms were restrained, excluding waters. An electrostatic and nonbonded cutoff of 8 Å, a Berendsen barostat, PME for long-range electrostatic interactions, and the SHAKE algorithm were applied to all calculations. Long timescale all-atom simulations were carried out in explicit water for the PD and analyzed for a better view of conformational changes in the CCMV capsid. Two simulations of NPT molecular dynamics were conducted for 2 ms in explicit water with different initial velocities, on constant temperature of 300K with Langevin thermostat and constant pressure of 1 bar with Berendsen barostat. The timestep used was 2 fs during the 2 ms simulation.

Production runs were performed in NPT ensemble with pressure set to 1 bar, as follows:

- $2 \,\upmu \hbox {s}$ on 300 K for T1, T2 and T3.
- $1.5 \,\upmu \hbox {s}$ on 300 K for TX.
- 600 ns on 350 K for T2, T3 and TX.

Implicit solvent simulations were performed with the OBC variant of the Generalized Born model (igb = 5)^36^, with no electrostatic cutoff, after heating the system to 350 K in 7 steps, increasing the temperature by 50 K in each step.

### Trajectory analysis

Trajectory analyses were carried out using the *cpptraj* software from the Amber14 package^37^, visualization was made with VMD^38^ and UCSF Chimera^39^.

RMSDs were calculated based on the input topology file to a reference frame of the coordinates both for $\mathrm {C}_\alpha$ and for the interface residues (iRMSD). Mean RMSD values were calculated for the whole trajectories in each case. Atomic positional fluctuations (RMSF), Connolly surface area^40^, secondary structures^41^, distance between the center of mass of monomers were determined. The interface surfaces for the dimers were calculated as the difference between the sum of the molecular surface areas for the separate chains and the molecular surface of the complex. Contacts between the atoms of the dimers were calculated with a distance cutoff of 7 Å. Binding energy was calculated with the MM-PBSA module of Amber14^42^.

Principal component analysis (PCA)^43^ was calculated for alpha-carbon atoms on trajectories. The first two principal components were computed and a covariance matrix was generated. The first two eigenvectors of the matrix correspond to PC1 and PC2. PCA values were linked to every atom and visualized with the NMWiz plugin in VMD^44^ where relative motions of the atoms are shown as arrows proportional with their values.

## Supplementary Information

Supplementary Information 1.

Supplementary Information 2.

Supplementary Information 3.

Supplementary Information 4.

Supplementary Movie 1.

Supplementary Movie 2.

Supplementary Movie 3.

Supplementary Movie 4.

Supplementary Movie 5.

Supplementary Movie 6.

## Data Availability

The datasets used and/or analysed during this study are available from the corresponding author on reasonable request.

## References

[1] Blöchliger N, Xu M, Caflisch A (2015). Peptide binding to a pdz domain by electrostatic steering via nonnative salt bridges. Biophys. J..

[2] Saglam AS, Wang DW, Zwier MC, Chong LT (2017). Flexibility vs preorganization: Direct comparison of binding kinetics for a disordered peptide and its exact preorganized analogues. J. Phys. Chem. B.

[3] Plattner N, Doerr S, Fabritiis G, Noé F (2017). Complete protein–protein association kinetics in atomic detail revealed by molecular dynamics simulations and Markov modelling. Nat. Chem..

[4] Paul F (2017). Protein-peptide association kinetics beyond the seconds timescale from atomistic simulations. Nat. Commun..

[5] Pan AC (2019). Atomic-level characterization of protein–protein association. Proc. Natl. Acad. Sci. U.S.A..

[6] Schreiber G (2002). Kinetic studies of protein–protein interactions. Curr. Opin. Struct. Biol..

[7] Lavelle L, Michel JP, Gingery M (2007). The disassembly, reassembly and stability of CCMV protein capsids. J. Virol. Methods.

[8] Díaz-Valle A, García-Salcedo YM, Chávez-Calvillo G, Silva-Rosales L, Carrillo-Tripp M (2015). Highly efficient strategy for the heterologous expression and purification of soluble cowpea chlorotic mottle virus capsid protein and in vitro pH-dependent assembly of virus-like particles. J. Virol. Methods.

[9] Hassani-Mehraban A, Creutzburg S, van Heereveld L, Kormelink R (2015). Feasibility of cowpea chlorotic mottle virus-like particles as scaffold for epitope presentations. BMC Biotechnol..

[10] Law-Hine D (2015). Reconstruction of the disassembly pathway of an icosahedral viral capsid and shape determination of two successive intermediates. J. Phys. Chem. Lett..

[11] Tresset G (2017). Two-dimensional phase transition of viral capsid gives insights into subunit interactions. Phys. Rev. Appl..

[12] Bereau T, Globisch C, Deserno M, Peter C (2012). Coarse-grained and atomistic simulations of the salt-stable cowpea chlorotic mottle virus (ss-ccmv) subunit 26–49: $$\beta$$-barrel stability of the hexamer and pentamer geometries. J. Chem. Theory Comput..

[13] Kortemme T, Baker D (2002). A simple physical model for binding energy hot spots in protein–protein complexes. Proc. Natl. Acad. Sci. U.S.A..

[14] Davis FP, Sali A (2005). PIBASE: A comprehensive database of structurally defined protein interfaces. Bioinformatics.

[15] Zhang L, Borthakur S, Buck M (2016). Dissociation of a dynamic protein complex studied by all-atom molecular simulations. Biophys. J ..

[16] Zhang L, Buck M (2013). Molecular simulations of a dynamic protein complex: Role of Salt-bridges and polar interactions in configurational transitions. Biophys. J ..

[17] Banerjee P, Bagchi B (2020). Dynamical control by water at a molecular level in protein dimer association and dissociation. Proc. Natl. Acad. Sci. U.S.A..

[18] Speir JA, Munshi S, Wang G, Baker TS, Johnson JE (1995). Structures of the native and swollen forms of cowpea chlorotic mottle virus determined by X-ray crystallography and cryo-electron microscopy. Structure.

[19] Chen J, Lansac Y, Tresset G (2018). Interactions between the molecular components of the cowpea chlorotic mottle virus investigated by molecular dynamics simulations. J. Phys. Chem. B.

[20] Bancroft JB, Rees MW, Johnson MW, Dawson JRO (1973). A salt-stable mutant of cowpea chlorotic mottle virus. J. Gen. Virol..

[21] Fox JM, Zhao X, Speir JA, Young MJ (1996). Analysis of a salt stable mutant of cowpea chlorotic mottle virus. Virology.

[22] Vangone A, Spinelli R, Scarano V, Cavallo L, Oliva R (2011). COCOMAPS: A web application to analyze and visualize contacts at the interface of biomolecular complexes. Bioinformatics.

[23] Salamanca Viloria J, Allega MF, Lambrughi M, Papaleo E (2017). An optimal distance cutoff for contact-based protein structure networks using side-chain centers of mass. Sci. Rep..

[24] Speir JA (2006). Enhanced local symmetry interactions globally stabilize a mutant virus capsid that maintains infectivity and capsid dynamics. J. Virol..

[25] Antal Z, Szoverfi J, Fejer S (2017). Predicting the initial steps of salt-stable cowpea chlorotic mottle virus capsid assembly with atomistic force fields. J. Chem. Inf. Model..

[26] Globisch C, Krishnamani V, Deserno M, Peter C (2013). Optimization of an elastic network augmented coarse grained model to study CCMV capsid deformation. PLoS ONE.

[27] Garmann RF, Comas-Garcia M, Gopal A, Knobler CM, Gelbart WM (2014). The assembly pathway of an icosahedral single-stranded rna virus depends on the strength of inter-subunit attractions. J. Mol. Biol..

[28] Chakravarty A, Reddy VS, Rao A (2020). Unravelling the stability and capsid dynamics of the three virions of brome mosaic virus assembled autonomously in vivo. J. Virol..

[29] Anandakrishnan R, Drozdetski A, Walker RC, Onufriev AV (2015). Speed of conformational change: Comparing explicit and implicit solvent molecular dynamics simulations. Biophys. J..

[30] Zlotnick A, Aldrich R, Johnson JM, Ceres P, Young MJ (2000). Mechanism of capsid assembly for an icosahedral plant virus. Virology.

[31] Schrödinger, LLC (2015). PyMOL—The PyMOL Molecular Graphics System, Version 1.8.

[32] Schafmeister C, Ross W, Romanovski V (1995). Leap.

[33] Jorgensen WL, Chandrasekhar J, Madura JD, Impey RW, Klein ML (1983). Comparison of simple potential functions for simulating liquid water. J. Chem. Phys..

[34] Maffucci I, Contini A (2016). Improved computation of protein–protein relative binding energies with the Nwat-MMGBSA method. J. Chem. Inf. Model..

[35] Duan Y (2003). A point-charge force field for molecular mechanics simulations of proteins based on condensed-phase quantum mechanical calculations. J. Comput. Chem..

[36] Onufriev A, Bashford D, Case DA (2004). Exploring protein native states and large-scale conformational changes with a modified generalized born model. Proteins Struct. Funct. Bioinform..

[37] Roe DR, Cheatham TE (2013). PTRAJ and CPPTRAJ: Software for processing and analysis of molecular dynamics trajectory data. J. Chem. Theory Comput..

[38] Humphrey W, Dalke A, Schulten K (1996). VMD: Visual molecular dynamics. J. Mol. Graph..

[39] Pettersen EF (2004). UCSF chimera—A visualization system for exploratory research and analysis. J. Comput. Chem..

[40] Connolly ML (1983). Analytical molecular surface calculation. J. Appl. Crystallogr..

[41] Kabsch W, Sander C (1983). Dictionary of protein secondary structure: Pattern recognition of hydrogen bonded and geometrical features. Biopolymers.

[42] Genheden S, Ryde U (2015). The MM/PBSA and MM/GBSA methods to estimate ligand-binding affinities. Expert Opin. Drug Discov..

[43] David CC, Jacobs DJ (2014). Principal component analysis: A method for determining the essential dynamics of proteins. Methods Mol. Biol. (Clifton, N.J.).

[44] Bakan A, Meireles LM, Bahar I (2011). ProDy: Protein dynamics inferred from theory and experiments. Bioinformatics.

