# Supplementary material for: Dynamic stability of salt stable cowpea chlorotic mottle virus capsid protein dimers and pentamers of dimers
Source: Sci Rep. 2022 Aug 22;12:14251. doi: 10.1038/s41598-022-18019-9 (PMC9395436; doi:10.1038/s41598-022-18019-9)
Supplement: Supplementary file 1 — Supplementary Information 1. [file 41598_2022_18019_MOESM1_ESM.pdf]

Supplementary material for the paper 'Dynamic stability of salt stable cowpea chlorotic mottle virus capsid protein dimers and pentamers of dimers'

Janos Szovérfi[1,2] **and Szilard N. Fejer**[2,3*]

[1]University Politehnica of Bucharest, Faculty of Chemical Engineering and Biotehnologies, 1-7 Gheorghe Polizu Street, 011061 Bucharest, Romania
[2]Provitam Foundation, 16 Caisului Street, 400487 Cluj-Napoca, Romania
[3]University of Pécs, Institute of Chemistry, 6 Ifjúság Street, Pécs, Hungary
[*]szilard.fejer@cantab.net

Supplementary Data

Trajectories

The supplementary data file contains trajectories for four dimer structures in Amber crd format: 4 parallel runs of the four dimer types each at 350 K in implicit solvent, one run of the four dimer types each at 350 K in explicit solvent, 2 µs simulations for the three native dimer types and the pentamer of dimers, 1.5 µs simulation for the non-native (TX) dimer, at 300 K in explicit solvent. The contact maps for the three dimers are also provided in three .csv files.

Movies

Supplementary Movie 1: Animation of the 2 µs NPT ensemble molecular dynamics simulations at 300 K for the T1 dimer.
Supplementary Movie 2: Animation of the 2 µs NPT ensemble molecular dynamics simulations at 300 K for the T2 dimer.
Supplementary Movie 3: Animation of the 2 µs NPT ensemble molecular dynamics simulations at 300 K for the T3 dimer.
Supplementary Movie 4: Animation of the 1.5 µs NPT ensemble molecular dynamics simulations at 300 K for the TX dimer.
Supplementary Movie 5: Animation showing the dissociation of the T2 dimer in implicit solvent molecular dynamics simulations at 350 K.
Supplementary Movie 6: Animation of the 2 µs NPT ensemble molecular dynamics simulations at 300 K for the pentamer of dimers.

The supplementary data can be downloaded from https://szilard.ro/files/ccmv-supplementary.zip.

Supplementary Figures

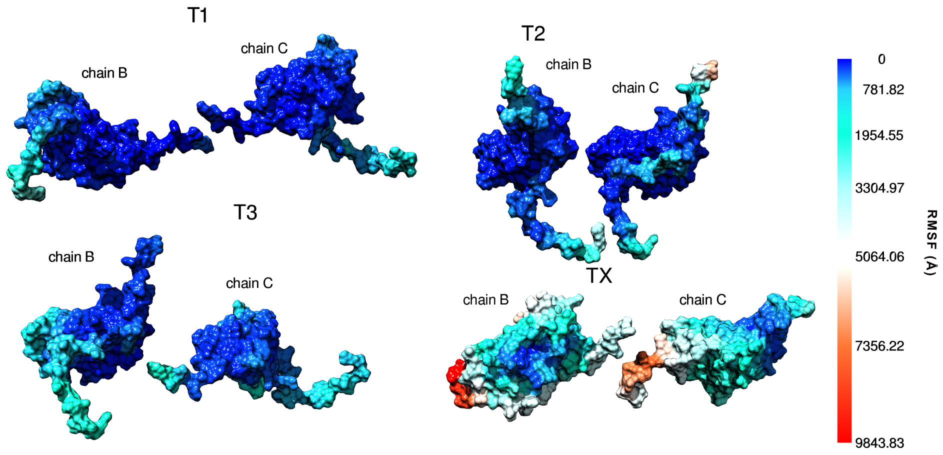

Figure S1. T1, T2, T3 and TX dimers with surface representations coloured by overall residue mobility during the long timescale simulations. The dimers are separated for a better view.

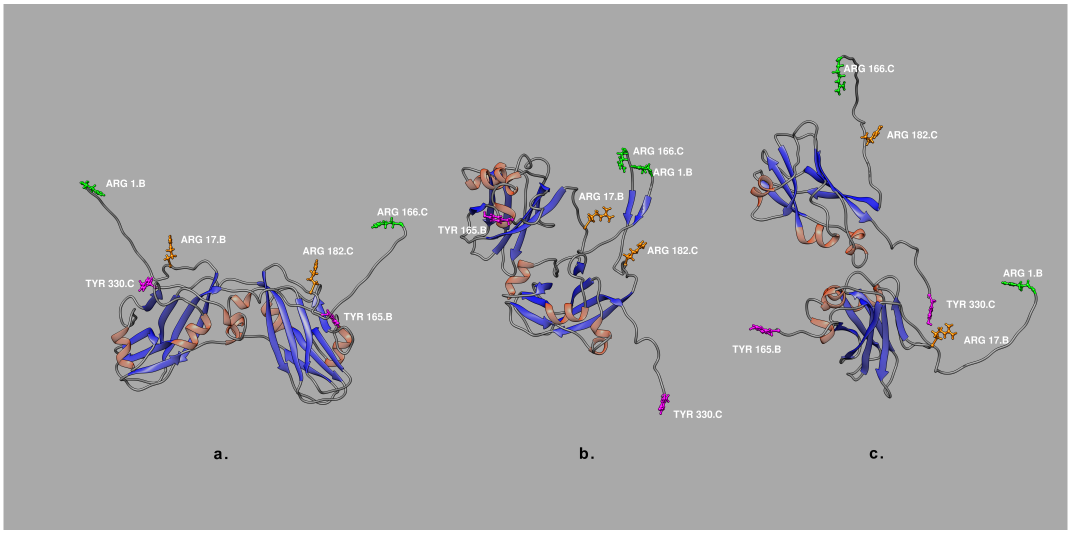

Figure S2. Cartoon representation coloured by secondary structure of T1 (**a.**), T2 (**b.**), T3 (**c.**) dimers. N-terminal (green), C-terminal (magenta) and the mutated arginine residues (orange) are highlighted.

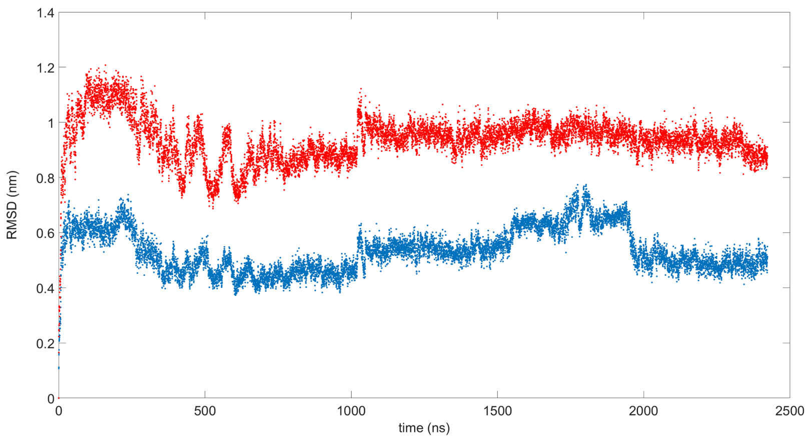

a.

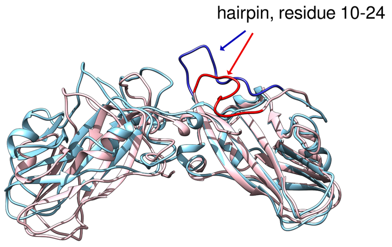

b.

Figure S3. **a.** C$_\alpha$ RMSD(red) and iRMSD (blue) for the 2.5 µs NPT explicit solvent for the T1 interface. **b.** The final frame of the simulation (red) and the structure at 1900 ns (blue) superimposed, where the temporary shift of the hairpin can be observed.

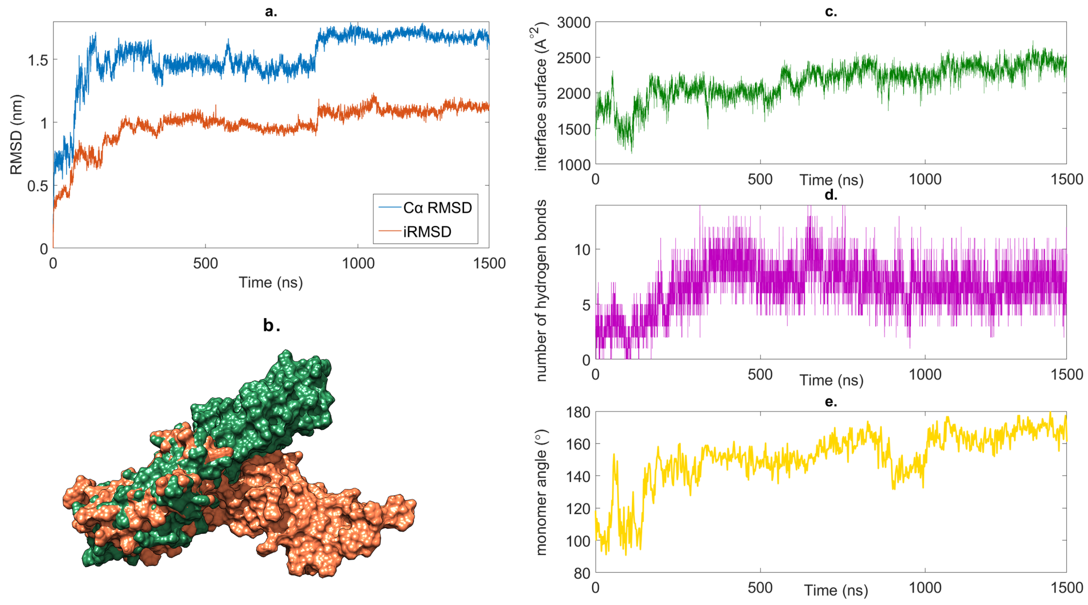

Figure S4. Analysis of the 1.5 µs NPT, explicit solvent simulation for the TX interface: **a.** Cα RMSD and iRMSD; **b.** initial (red) and final (green) structures of TX superimposed with surface representation; **c.** change in the interface surface; **d.** number of hydrogen bonds; **e.** variation of the angle between the monomers of the protein.

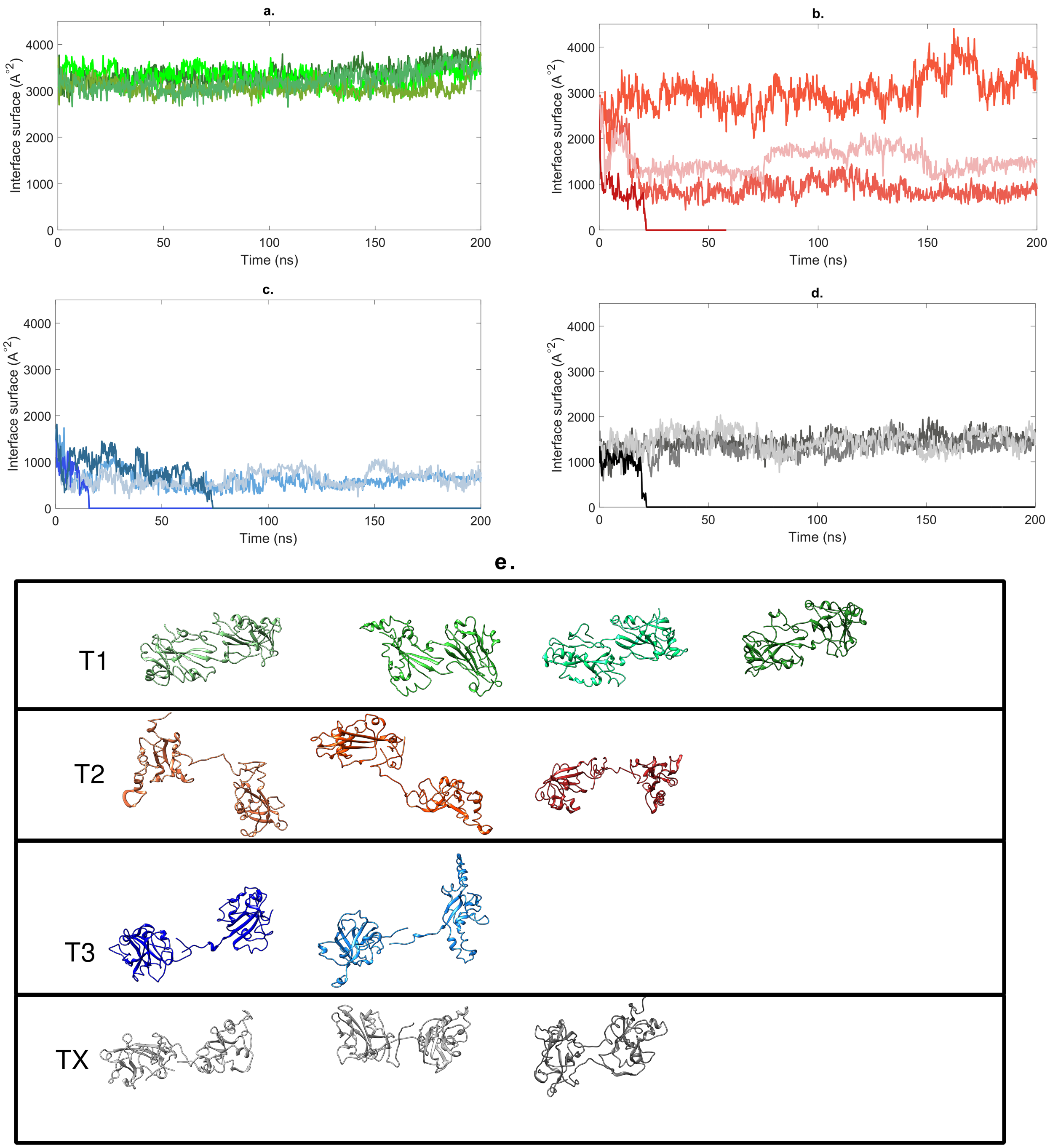

Figure S5. Variation of the interface surface for T1 (**a.**), T2 (**b.**), T3 (**c.**) and TX (**d.**) in 4 parallel runs for 200 ns implicit solvent simulation on 350 K. **e.** Graphical representations of the undissociated structures from the 4 parallel runs (T1-green, T2-red, T3-blue, TX-green) at the end of the 200 ns simulations. In total, 12 structures remained attached to each other, out of which 8 structures are loosely associated.

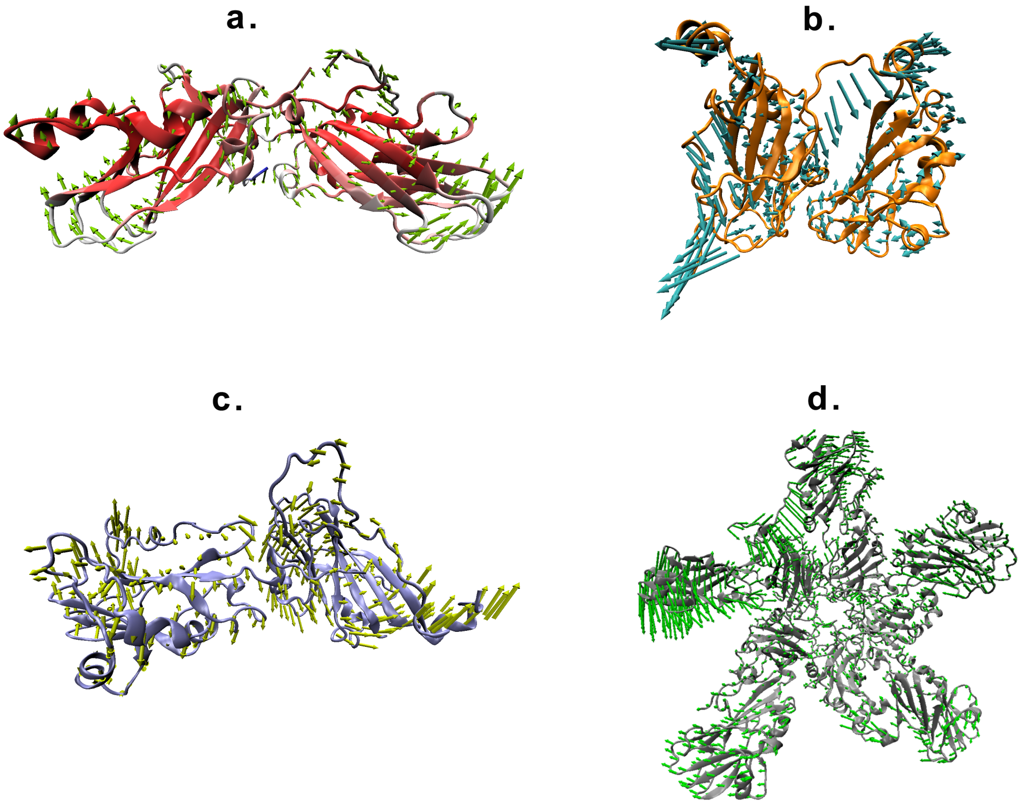

Figure S6. Relative motions of the C_α atoms shown as arrows aligned to the average structure of 2 μs NPT ensemble simulation for T1 (**a.**), T2 (**b.**), T3 (**c.**) and PD (**d.**).

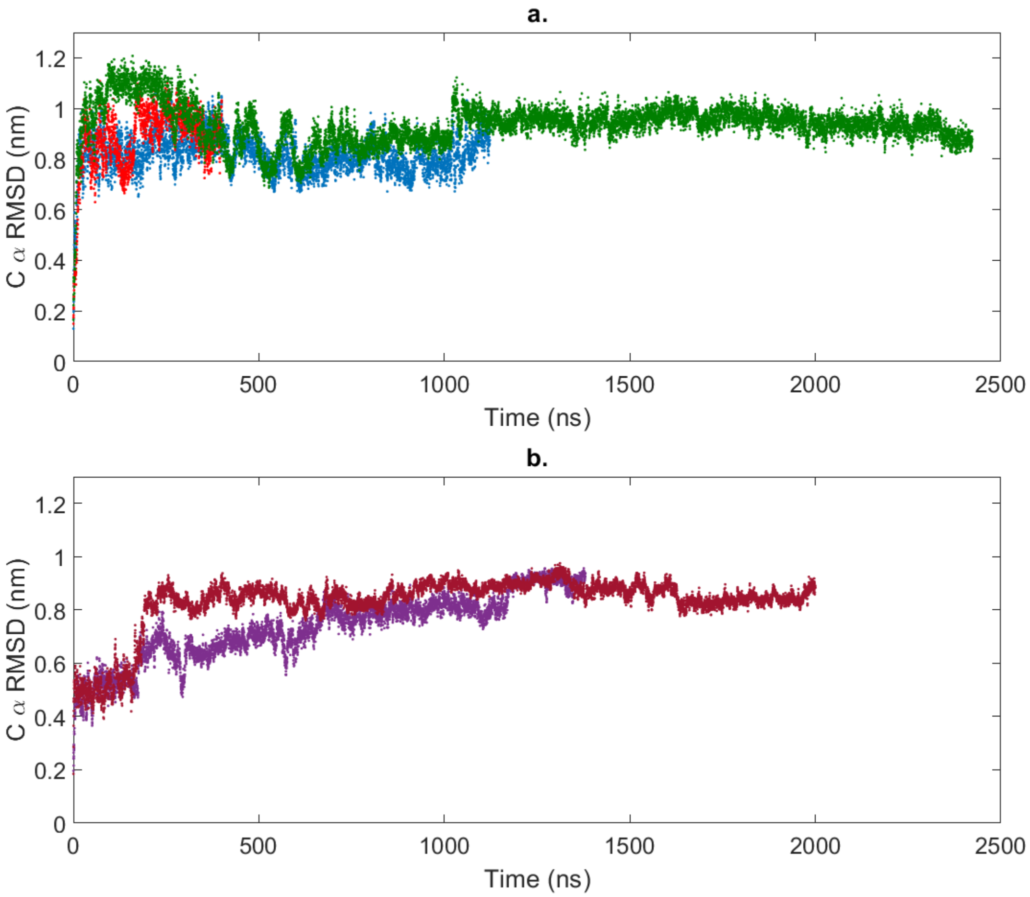

Figure S7. C_α RMSD of parallel simulations in NPT explicit solvent for the T1 interface (**a.**) and for the T2 interface (**b.**)
